# Complete mitochondrial genome of *Pisaura bicornis* (Araneae: Pisauridae) and its phylogenetic implications

**DOI:** 10.1080/23802359.2019.1667921

**Published:** 2019-09-24

**Authors:** Shu-Yan Yan, Kang-Kang Xu, Da-Xing Yang, Can Li, Wen-Jia Yang

**Affiliations:** Guizhou Provincial Key Laboratory for Rare Animal and Economic Insect of the Mountainous Region, College of Biology and Environmental Engineering, Guiyang University, Guiyang, China

**Keywords:** *Pisaura bicornis*, Pisauridae, mitogenome

## Abstract

The complete mitochondrial genome of *Pisaura bicornis* (GenBank accession number MN296112) is 15,281 bp in length and contains 13 protein-coding genes (PCGs), 22 transfer RNA genes (tRNAs), 2 ribosomal RNA genes, and a putative control region. The overall base composition of this genome is A (35.65%), C (8.12%), G (13.47%) and T (42.76%), demonstrating an obvious bias of high AT content (73.2%). ATT, ATA, TTG were initiation codons and TAA, TAG, and T were termination codons. Ten tRNAs (*trnD*, *trnF*, *trnG*, *trnH*, *trnK*, *trnP*, *trnR*, *trnT*, *trnY*, and *trnE*) lacked the TΨC arm stem, while three tRNAs (*trnA*, *trnS_1_*, and *trnS_2_*) lost the dihydrouracil (DHU) arm. Phylogenetic tree based on 13 PCGs showed that *P. bicornis* is closely related to *Dolomedes angustivirgatus* and belongs to Pisauridae.

The nursery-web spider *Pisaura bicornis* belongs to the family Pisauridae, which comprises more than 300 species in 48 genera around the world (Sierwald 1997; Platnick 2015). Most of these spiders are carnivores and often live in farmland or near streams (Zhang 2000). In this study, adult species of *P. bicornis* were collected from Maolan Nature Reserve in Libo county (N25°20′, E107°55′), Guizhou Province, China, and deposited in the spider specimen room of Guiyang University with an accession number GYU-GZML-06.

The complete mitogenome of *P. bicornis* (GenBank accession number MN296112) is 15,281 bp in length and contains 13 protein-coding genes (PCGs), 22 transfer RNA genes (tRNAs), two ribosomal RNA genes (rRNAs), and a putative control region. The gene content and arrangement of *P. bicornis* are similar to those found in previously determined spider mitogenomes (Li et al. 2016; Yang et al. 2019). Four PCGs (*nad5*, *nad4*, *nad4L*, and *nad1*), nine tRNAs (*trnY*, *trnC*, *trnL_2_*, *trnF*, *trnH*, *trnP*, *trnL_1_*, *trnV*, and *trnQ*), and two rRNAs (*rrnL* and *rrnS*) were encoded on the light strand (N-strand). Nine PCGs (*nad2, cox1, cox2, cox3, atp6, atp8, nad3, nad6*, and *cytb*) and thirteen tRNAs (*trnM*, *trnW*, *trnK*, *trnD*, *trnG*, *trnN*, *trnA*, *trnS_1_*, *trnR*, *trnE*, *trnI*, *trnS_2_*, and *trnT*) were encoded on the heavy strand (J-strand). The overall base composition of *P. bicornis* mitogenome was A (35.65%), C (8.12%), G (13.47%) and T (42.76%), with a high AT bias of 73.2%. The AT-skew and GC-skew of this mitogenome were −0.091 and 0.248, respectively.

Gene overlaps were found in 23 locations and involved a total of 214 bp. The longest overlap is 45 bp in length and located between *nad4* and *nad4L*. There are 8 intergenic spacer regions comprising a total of length of 49 bp and the largest spacer (15 bp) resided between *trnP* and *nad6*. The length of 22 tRNAs ranged from 48 bp (*trnK*) to 70 bp (*trnI*), The A + T content ranged from 69.09% (*trnR*) to 88.14% (*trnC*). Thirteen tRNAs lacked the potential to form the cloverleaf secondary structure. Ten of them (*trnD*, *trnF*, *trnG*, *trnH*, *trnK*, *trnP*, *trnR*, *trnT*, *trnY*, and *trnE*) lacked the TΨC arm stem, whereas three tRNAs (*trnA*, *trnS_1_*, and *trnS_2_*) lost the dihydrouracil (DHU) arm. The *rrnL* was located between *trnL_1_* and *trnV* and the *rrnS* was determined between *trnV* and *trnQ*. The length of *rrnL* and *rrnS* is 1,020 bp and 697 bp, and their A + T content were 82.55% and 82.07%, respectively. The control region was 1,638 bp in length with an A + T content of 77.84% and located between the *trnQ* and *trnM*.

Eleven PCGs started with a typical ATN start codons (ATT and ATA), and the remaining two PCGs (*cox1* and *cox3*) began with TTG. Ten PCGs terminated with TAA, two (*nad1* and *nad2*) terminated with TAG, whereas *nad4L* terminated with an incomplete stop codon T. Based on the concatenated amino acid sequences of 13 PCGs, the neighbor-joining method was used to construct the phylogenetic relationship of *P. bicornis* with 16 other known spiders. The result showed that *P. bicornis* is closely related to *Dolomedes angustivirgatus* and belongs to Pisauridae (Figure 1).

**Figure 1.**
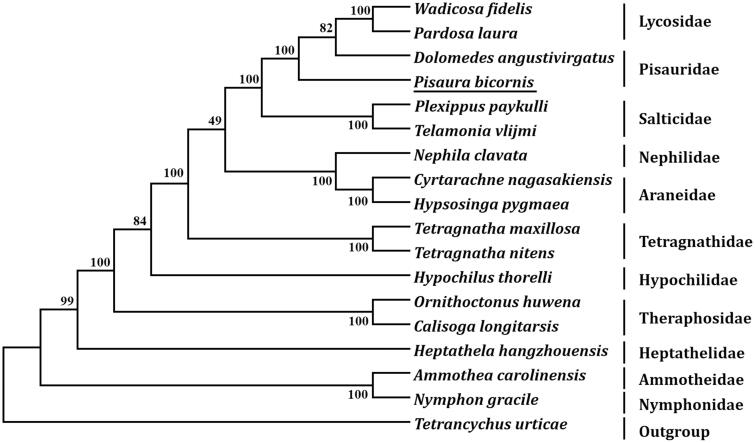
Phylogenetic tree showing the relationship between *Pisaura bicornis* and 16 other spiders based on neighbor-joining method. GenBank accession numbers used in the study are the following: *Ammothea carolinensis* (GU065293), *Calisoga longitarsis* (NC_010780), *Cyrtarachne nagasakiensis* (KR259802), *Dolomedes angustivirgatus* (NC_031355), *Heptathela hangzhouensis* (NC_005924), *Hypochilus thorelli* (EU523753), *Hypsosinga pygmaea* (KR259803), *Nephila clavata* (AY452691), *Nymphon gracile* (NC_008572), *Ornithoctonus huwena* (AY309259), *Pardosa laura* (KM272948), *Pisaura bicornis* (MN296112), *Plexippus paykulli* (KM114572), *Telamonia vlijmi* (NC_024287), *Tetragnatha maxillosa* (KP306789), *Tetragnatha nitens* (KP306790), *Tetrancychus urticae* (EU345430), and *Wadicosa fidelis* (NC_026123). *T. urticae* was used as an outgroup. Spider determined in this study was underlined.

## References

[CIT0001] Li C, Wang ZL, Fang WY, Yu XP 2016 The complete mitochondrial genome of the orb-weaving spider *Neoscona theisi* (Walckenaer) (Araneae: Araneidae). Mitochondr DNA A DNA Mapp Seq Anal. 27:4035–4036. 10.3109/19401736.2014.1003831 25629467

[CIT0002] Platnick NI 2015 The world spider catalog, Version 15.0. American Museum of Natural History. http://research.amnh.org/iz/spiders/catalog/.

[CIT0003] Sierwald P 1997 Phylogenetic analysis of Pisaurine nursery web spiders, with revisions of Tetragonophthalma and Perenethis (Araneae, Lycosoidea, Pisauridae. J Arachnol. 25:361–407.

[CIT0004] Yang WJ, Xu KK, Liu Y, Yang DX, Li C 2019 Complete mitochondrial genome and phylogenetic analysis of *Argiope perforata* (Araneae: Araneidae). Mitochondr DNA B. 4:1963–1964.

[CIT0005] Zhang JX 2000 Taxonomy studies on Chinese spiders the genus Pisaura (Araneae: Pisauridae. Acta Arachnol Sin. 9:1–9.

